# Genetic evidence for a causal link between gut microbiota and arterial embolism and thrombosis: a two-sample Mendelian randomization study

**DOI:** 10.3389/fmicb.2024.1396699

**Published:** 2024-06-18

**Authors:** Yong-Bin Shi, Hong-Lin Dong, Wen-Kai Chang, Yan Zhao, Hai-Jiang Jin, Jun-Kai Li, Sheng Yan

**Affiliations:** ^1^Department of Vascular Surgery, Second Hospital of Shanxi Medical University, Taiyuan, China; ^2^Department of Vascular Surgery, First Hospital of Shanxi Medical University, Taiyuan, China

**Keywords:** arterial embolism and thrombosis, gut microbiota, Mendelian randomization, causal relationship, single-nucleotide polymorphism

## Abstract

**Background:**

Previous research has hinted at a crucial link between gut microbiota and arterial embolism and thrombosis, yet the causal relationship remains enigmatic. To gain a deeper understanding, we aimed to comprehensively explore the causal relationship and elucidate the impact of the gut microbiota on the risk through a two-sample Mendelian randomization (MR) study.

**Methods:**

Genetic instrumental variables for gut microbiota were identified from a genome-wide association study (GWAS) of 18,340 participants. Summary statistics for IBS were drawn from a GWAS including 1,076 cases and 381,997 controls. We used the inverse-variance weighted (IVW) method as the primary analysis. To test the robustness of our results, we further performed the weighted median method, MR-Egger regression, and MR pleiotropy residual sum and outlier test.

**Results:**

We identified three bacterial traits that were associated with the risk of arterial embolism and thrombosis: odds ratio (OR): 1.58, 95% confidence interval (CI): 1.08–2.31, *p* = 0.017 for genus *Catenibacterium*; OR: 0.64, 95% CI: 0.42–0.96, *p* = 0.031 for genus *Dialister*; and OR: 2.08, 95% CI: 1.25–3.47, *p* = 0.005 for genus *Odoribacter*. The results of sensitivity analyses for these bacterial traits were consistent (*P*<0.05).

**Conclusion:**

Our systematic analyses provided evidence to support a potential causal relationship between several gut microbiota taxa and the risk of arterial embolism and thrombosis. More studies are required to show how the gut microbiota affects the development of arterial embolism and thrombosis.

## Introduction

Thrombosis, characterized by localized blood clotting, can occur within either the arterial or venous circulation, exerting significant medical implications (Mackman, 2008). Acute arterial thrombosis serves as the primary etiology for the majority of myocardial infarctions (heart attacks) and approximately 80% of strokes, constituting the leading cause of mortality in developed nations (Mackman, 2008; GBD Collaborators, 2021). Furthermore, acute thrombotic diseases collectively represent the foremost cause of global mortality and contribute significantly to the global disease burden, as assessed by disability-adjusted life years (Wendelboe and Raskob, 2016; St Germain et al., 2023). Given the substantial morbidity and mortality linked to acute thrombosis, there is an urgent imperative to enhance our comprehension of its pathogenesis and establish effective prevention strategies.

The human gut harbors trillions of microbial cells, comprising a vital component of our healthy physiological ecosystem, encompassing bacteria, fungi, archaea, and viral communities, collectively referred to as the “microbiota” (Tang et al., 2017). Alterations in the composition and dysregulation of the gut microbiota, known as dysbiosis, have been implicated in the onset of various diseases (Tang et al., 2017). In recent years, there has been a surge of interest in the interaction between the gut microbiota and the host. A mounting body of evidence indicates that the gut microbiota plays a pivotal role in human health and disease, encompassing arterial embolism and thrombosis (Koren et al., 2011; Tang et al., 2017, 2019). For example, some studies have reported cases implicating *Dialister* in the occurrence of arterial embolism and thrombosis (Pierre Lepargneur et al., 2006; Koren et al., 2011). However, these observational findings are susceptible to unmeasured confounding factors and may not accurately depict the relationship between gut microbiota and arterial embolism and thrombosis. Furthermore, reverse causation could introduce bias if alterations or cessation of the gut microbiota were influenced by the onset or progression of arterial embolism and thrombosis.

Mendelian randomization (MR) studies have been widely applied in etiological research for diseases in recent years. In the absence of randomized controlled trials, MR is the most persuasive strategy for exploring causality between exposure and outcomes (Zuccolo and Holmes, 2017). MR mimics randomized controlled trials by using single-nucleotide polymorphisms (SNPs) related to exposure as instrumental variables (IVs) (Hemani et al., 2018). This IV approach replicates randomization as SNPs are randomly allocated to offspring at conception, largely avoiding confounding factors, and individual factors such as sex and age are unlikely to bias causal effects(Burgess et al., 2013; Richmond and Davey Smith, 2022). Similarly, due to the formation of genotypes occurring before disease onset, errors related to reverse causality in MR studies are also unlikely (Burgess et al., 2013). In this study, we chose 196 gut microbiotas as exposures and arterial embolism as the outcome for MR analysis. Our objective was to investigate the potential causal relationship between gut microbiota, aiming to establish a theoretical foundation for further research into the intricate mechanisms and risk factors associated with arterial embolism and thrombosis.

## Materials and methods

### Study design

An effective MR study should adhere to three assumptions: (1) a robust and strong correlation between the IVs and the exposure factor; (2) independence between the IVs and confounding factors that influence the “exposure-outcome” relationship; and (3) genetic variation can only affect the occurrence of the outcome through the exposure factor and not through other pathways (Davies et al., 2018).

**Figure 1:**
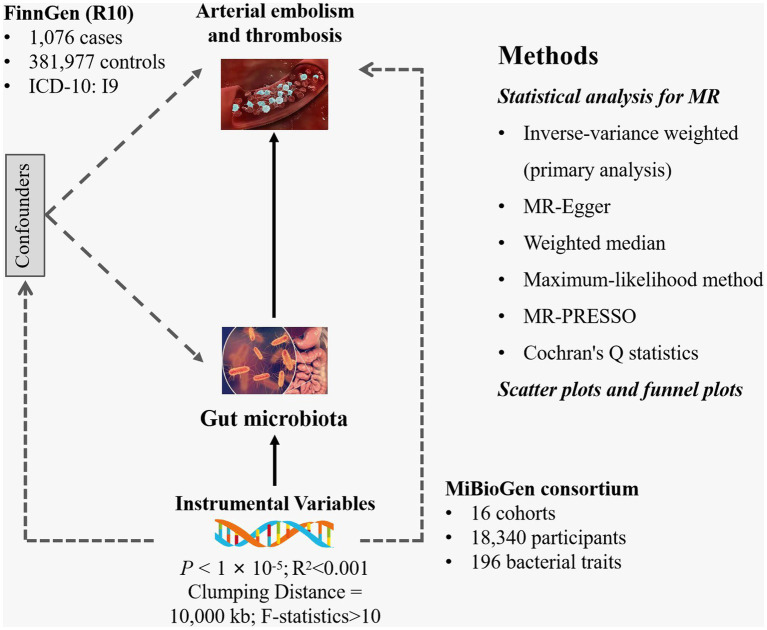
Study design of the present MR study of the associations between gut microbiota and arterial embolism and thrombosis of the lower extremity artery. MR, Mendelian randomization; SNP, single-nucleotide polymorphism.

Gut microbiota was selected as the exposure, while arterial embolism and thrombosis of the lower extremity artery served as the outcome. All statistics involved in the analysis were derived from publicly available genome-wide association studies (GWASs) (Supplementary Table S1). SNPs associated with gut microbial taxa and gut microbial metabolites were extracted as IVs. Based on the summary-level data from GWAS of gut microbiota and arterial embolism and thrombosis, we conducted a two-sample MR analysis. The flowchart of the study is shown in Figure 1.

### Exposure data of gut microbiota

The comprehensive GWAS summary statistics on the microbiome are mainly derived from the international consortium MiBioGen (Kurilshikov et al., 2021). This consortium incorporates data from 16 cohorts in different countries, including 18,340 participants with 24S rRNA gene sequencing profiles and genotyping data (Kurilshikov et al., 2021). The large-scale, multi-ethnic, whole-genome meta-analysis examines the association between common human genetic variations and the gut microbiome (Kurilshikov et al., 2021). The summary data include 211 gut microbial taxa. Relevant GWAS data can be accessed at https://mibiogen.gcc.rug.nl/. This represents the largest genome-wide meta-analysis conducted to date on the microbiome (Kurilshikov et al., 2021).

### Outcome data of arterial embolism and thrombosis of lower extremity artery

The summary data for arterial embolism and thrombosis of the lower extremity artery were retrieved from FinnGen’s online database (Kurki et al., 2023). This is a unique study combining genotype data from the Finnish Biobank and digital healthcare data from the Finnish Health Registry for both participants aged 18 years and older residing in Finland adjusting for age, sex, genetic relatedness, genotyping batch, and principal components (Kurki et al., 2023). Within this GWAS for Arterial embolism and thrombosis of lower extremity artery, total 383,053 European participants were included, with 381,977 controls and 1,076 Arterial embolism and thrombosis of lower extremity artery cases which were diagnosed according to the International Classification of Diseases codes (ICD-10: I9) (Kurki et al., 2023).

### Genetic instruments selection and harmonization

We first removed 15 bacterial traits without a specific name, leaving 196 bacterial traits, including 9 phylum, 16 class, 20 order, 32 family, and 119 genus (Liu et al., 2022). To guarantee the reliability and precision of the outcomes, the SNPs underwent a thorough quality assessment to yield compliant IVs. The principles guiding the selection of SNPs were outlined as follows: Firstly, the SNPs must exhibit a strong correlation with the exposure; second, the SNPs must be unrelated to any confounders; and finally, the SNPs must be associated with the outcomes mediated by the exposures (Davies et al., 2018). To achieve a more comprehensive result, a genome-wide statistical significance threshold of *p* < 1 × 10^−5^ was chosen for selecting eligible IVs (Liu et al., 2022). Then, to eliminate linkage disequilibrium (LD), a clumping method with r^2^ = 0.001 and kb = 10,000 was applied. Finally, the F-statistics were calculated to assess the strength of the selected SNPs using the formula: R^2^ = 2 MAF (1 − MAF) × β^2^, F = R^2^ (n-k-1) / k(1-R^2^) (Palmer et al., 2012; Kamat et al., 2019). In this formula, “MAF” is the minor allele frequency of SNPs used as IVs, R^2^ is the fraction of variability explained by each SNP, N is the GWAS sample size, and K is the number of SNPs. MAF is the A F statistic of 10 indicates that there is no convincing evidence of instrument bias (Georgakis et al., 2019).

### Statistical analysis

We used the inverse-variance weighted (IVW) method as the primary analysis. Then we applied a range of sensitivity analyses to assess the robustness of the IVW findings against potential violations, including MR-Egger, weighted median, maximum-likelihood method, and MR pleiotropy residual sum and outlier (MR-PRESSO) analyses. IVW utilizes a meta-analytic approach to pool the Wald estimates for each SNP, which is the most popular and reliable MR analysis technique (Burgess et al., 2013). MR-Egger is used to account for the uncorrelated pleiotropy of SNPs as IVs (Bowden et al., 2015). Weighted median method ensures reliable causal effect estimates if over half its weight derives from valid IVs (Bowden et al., 2016). The maximum-likelihood method was used, which may display a more credible association result when measurement error existed in the SNP-exposure effects (Hemani et al., 2018). For sensitivity analysis, Cochran’s Q statistic was utilized to assess heterogeneity, with a *p*-value of <0.05 regarded as significant heterogeneity (Burgess et al., 2017). If there is existing heterogeneity, random-effect IVW is performed instead of fixed-effect IVW (Burgess et al., 2017). MR-Egger is used to account for the uncorrelated pleiotropy of SNPs as IVs (Bowden et al., 2015). To exclude the effect of horizontal pleiotropy, MR-Egger regression was used to assess pleiotropy through the intercept term. An intercept *p*-value of <0.05 implied the presence of horizontal pleiotropy (Bowden et al., 2015). Moreover, the MR-PRESSO method was performed to remove possible horizontal pleiotropic outliers. After removing the outliers, the data were subsequently re-analyzed (Verbanck et al., 2018). Additionally, we used scatter plots and funnel plots to detect whether there were existing outliers and evident heterogeneity.

The association between gut microbiota and arterial embolism and thrombosis was represented using odds ratios (ORs) along with 95% confidence intervals (CIs). A *p*-value of <0.05 was considered the significance threshold. All the analyses were conducted by applying packages “TwoSampleMR,” “MRPRESSO,” and “MendelianRandomization” in R version 4.1.3. The analysis codes are shown in Supplementary Table S1.

## Results

### Selection of instrumental variables

**Figure 2:**
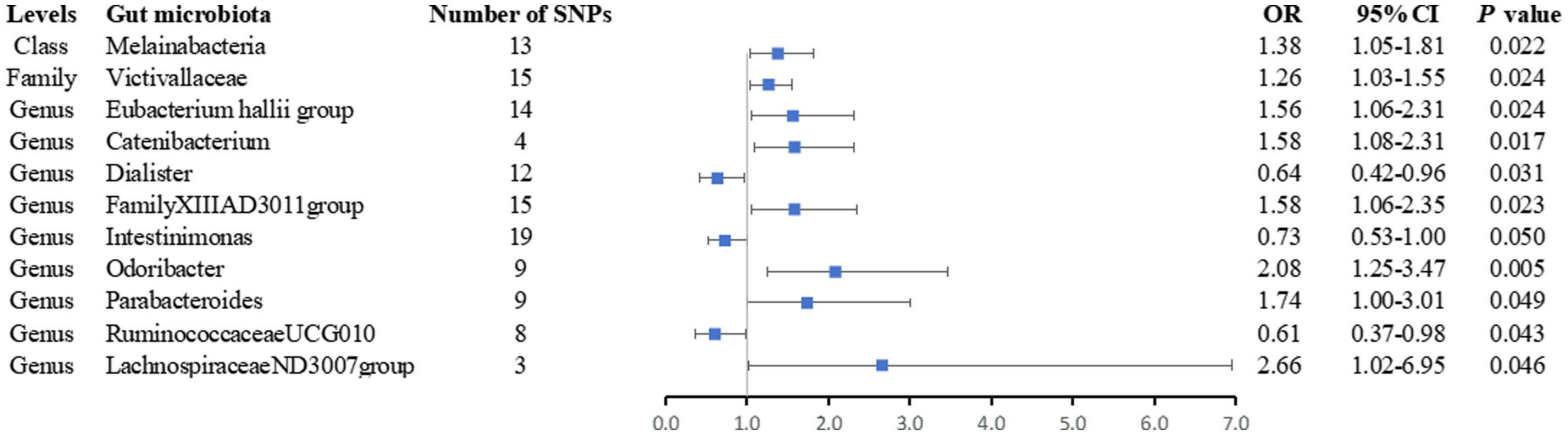
Forest plot of the associations between genetically determined 11 bacterial traits and arterial embolism and thrombosis of lower extremity artery risk. CI, confidence interval; OR, odds ratio; SNP, single-nucleotide polymorphism.

After removing palindromic SNPs from a large-scale GWAS, we initially identified 2,699 SNPs related to 196 gut microbiota (with a locus-wide significance level of *p* < 1 × 10^−5^) (Supplementary Tables S2, S3). For IVs used for gut microbiota, the *F*-statistics ranged from 21.8 to 142.0, which were all above 10, suggesting weak instrument bias was less likely (Supplementary Table S2). We observed statistically significant evidence for a potential causal association between 11 gut microbiota and the risk of AS using the IVW method, including one class, one family, and nine genera. The details of SNPs used for bacterial traits are listed in Supplementary Table S3, and the results of the associations between the 11 bacterial traits and the risk of arterial embolism and thrombosis are presented in Figure 2 and Supplementary Table S4.

**Figure 3:**
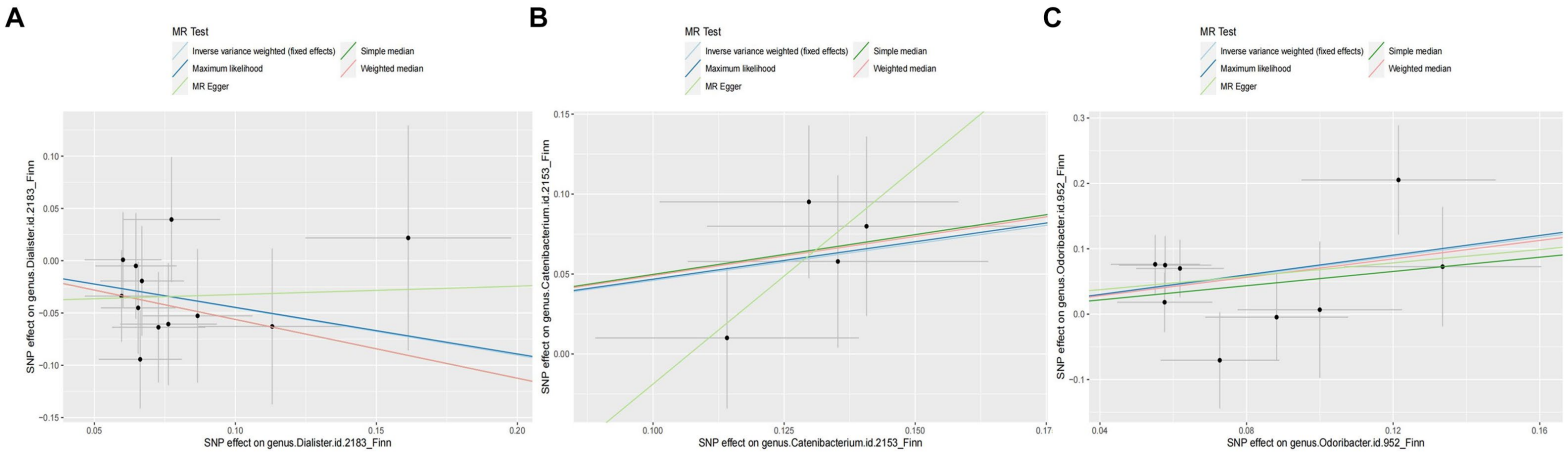
Scatter plot of the associations of genetic variants with three bacterial traits and the risk of arterial embolism and thrombosis. MR, mendelian randomization; SNP, single-nucleotide polymorphism. **(A)** SNP effect on genus *Dialister*; **(B)** SNP effect on genus *Catenibacterium*; **(C)** SNP effect on genus *Odoribacter*.

Overall, we found that genus *Catenibacterium* was positively associated with the risk of arterial embolism and thrombosis of the lower extremity artery by the IVW method (OR: 1.58, 95% CI: 1.08–2.31, *p* = 0.017) (Figure 3). Using alternative MR methods, we observed similar results. For genus *Catenibacterium*, a similar effect estimate was obtained in the weighted median method (OR: 1.66, 95% CI: 1.05–2.62, *p* = 0.031) and the maximum-likelihood method (OR: 1.60, 95% CI: 1.08–2.37, *p* = 0.020). MR-PRESSO test did not detect any outliers, and the association estimate was similar (OR: 1.58, 95% CI: 1.20–2.08, *p* = 0.046). Little evidence of directional pleiotropy was detected by MR-Egger regression (*P _intercept_* = 0.352).

In addition, we also observed that genus *Odoribacter* was positively associated with the risk of arterial embolism and thrombosis of the lower extremity artery by IVW method (OR: 2.08, 95% CI: 1.25–3.47, *p* = 0.005) (Figure 3). The sensitivity analysis results were consistent (OR: 2.30, 95% CI: 1.13–4.69, *p* = 0.021 for weighted median method; OR: 2.14, 95% CI: 1.26–3.62, *p* = 0.005 for maximum-likelihood method). MR-PRESSO test found no potential outlier, and the association remained statistically significant (OR: 2.08, 95% CI: 1.25–3.49, *p* = 0.005). MR-Egger regression did not suggest directional pleiotropy (*P _intercept_* = 0.803).

However, genus *Dialister* was negatively associated with the risk of arterial embolism and thrombosis of the lower extremity artery by IVW (OR: 0.64, 95% CI: 0.42–0.96, *p* = 0.031) (Figure 3). Similar associations were observed using the weighted median method (OR: 0.57, 95% CI: 0.34–0.96, *p* = 0.035), maximum-likelihood method (OR: 0.64, 95% CI: 0.43–0.98, *p* = 0.038), and MR-PRESSO test (OR: 0.64, 95% CI: 0.47–0.85, *p* = 0.012). The intercepts of MR-Egger regression did not significantly deviate from zero for them (*P*_intercept_ = 0.536).

Finally, we noticed that the rest of the six bacterial traits were associated with a higher risk of arterial embolism and thrombosis of the lower extremity artery in the IVW method (OR: 1.38; 95% CI: 1.05–1.81; *p* = 0.022 for class Melainabacteria; OR: 1.26; 95% CI: 1.03–1.55; *p* = 0.024 for family Victivallaceae; OR: 1.56; 95% CI: 1.06–2.31; *p* = 0.024 for genus *Eubacterium hallii* group OR: 1.58; 95% CI: 1.06–2.35; *p* = 0.023 for genus *FamilyXIIIAD3011group*; OR: 1.74; 95% CI: 1.00–3.01; *p* = 0.049 for genus *Parabacteroides;* and OR: 2.66; 95% CI: 1.02–6.95; *p* = 0.046 for genus *LachnospiraceaeND3007group*) while two were associated with a lower risk (OR: 0.73; 95% CI: 0.53–1.00; *p* = 0.050 for genus *Intestinimonas* and OR: 0.61; 95% CI: 0.37–0.98; *p* = 0.012 for genus *RuminococcaceaeUCG010*).

## Discussion

To our knowledge, this is the first MR study that integrates gut microbiota, arterial embolism, and thrombosis of the lower extremity artery. This two-sample MR study identified a total of 11 bacterial taxa, including class *Melainabacteria*, *family* Victivallaceae, genus *Eubacterium hallii group*, genus *Catenibacterium*, *Dialister*, *FamilyXIIIAD3011 group*, *Intestinimonas*, *Odoribacter*, *Parabacteroides*, *RuminococcaceaeUCG010*, and *LachnospiraceaeND3007* group might have a causal relationship with the risk of arterial embolism and thrombosis of the lower extremity artery. However, sensitivity analyses using different MR methods and restricted IV sets demonstrated three bacterial taxa, *Catenibacterium*, *Dialister*, and *Odoribacter*, associated with the risk of arterial embolism and thrombosis of the lower extremity artery. These results will contribute to further exploration of the roles of gut microbiota and blood metabolites in the process of arterial embolism and thrombosis of the lower extremity artery. Furthermore, they provide a reference for future intervention measures and the development of potential therapeutic targets.

A two-sample MR study found 17 bacterial traits and their association with the risk of venous thromboembolism, including family Victivallaceae (Wang et al., 2024), which was consistent with our study’s finding of an increased risk of arterial embolism. However, another two-sample MR study found seven causal associations between genetic liability in the gut microbiota and lower extremity deep vein thrombosis (LEDVT) (Hirai et al., 2022). These findings did not align with the results of our study, which could be attributed to differences in the study populations.

The genus *Dialister* has also been identified as a protective factor against arterial embolism and thrombosis in the lower extremity artery. For instance, dysbiosis in *Dialister* has been associated with various diseases, including thrombosis (Pierre Lepargneur et al., 2006; Xu et al., 2024). Another study has also identified that an increase in Actinobacteria would decrease the risk of thrombosis (Yang et al., 2022). In addition, the abundance of *Dialister* showed significant alterations in healthy controls and cardiovascular and cerebrovascular diseases (Ji et al., 2017; Chang et al., 2021; Han et al., 2021). The genus *Dialister* may produce molecules with immunoregulatory and anti-inflammatory functions, such as short-chain fatty acids (SCFAs), indoles and their derivatives, and secondary bile acids. These molecules could potentially regulate the immune system by inhibiting the activity of immune cells, thus modulating the intensity and direction of immune responses, which may mitigate the risk of arterial embolism and thrombosis (Sun et al., 2022). The potential causal relationship observed in this study between Dialister and arterial embolism and thrombosis underscores the significance of Dialister in the development of these conditions.

The bacterial taxa genus *Catenibacterium* and *Odoribacter* were found to be positively associated with the risk of arterial embolism and thrombosis in this study. However, to date, there have been no studies reporting alterations in the genus *Catenibacterium* in patients with arterial embolism and thrombosis. We noticed an increasing trend for the genus *Catenibacterium* and *Odoribacter* in stroke cases compared to healthy controls (Li et al., 2019; Yu et al., 2023; Yuan et al., 2023). It has been shown that *Catenibacterium* and *Odoribacter* could be linked to obesity (Pinart et al., 2021), which is known to increase the risk of arterial embolism. While this study is the first to suggest a potential causal relationship between the genera *Catenibacterium* and *Odoribacter* and the risk of arterial embolism and thrombosis, further research is warranted to elucidate the underlying biological mechanisms.

There are potential explanations for how the gut microbiota influences arterial embolism and thrombosis. The intestinal microbiota is segregated from the host by the mucus layer and a specialized epithelial lining (Johansson et al., 2011; Duerr and Hornef, 2012). The epithelium must meet precise criteria to shield the host from foreign antigens while simultaneously permitting substances to enter the portal circulation for energy metabolism and sufficient nutrient provision (Lingaraju et al., 2015). The gut–vascular barrier, in addition to the epithelial barrier, must also meet these requirements (Spadoni et al., 2015; Thevaranjan et al., 2017). It is noteworthy that the gut microbiota not only influences the permeability of the intestinal vasculature but also has the capability to induce the development of complex capillary networks and lacteals within the villus structures of the small intestine, facilitating nutrient absorption (Stappenbeck et al., 2002; Reinhardt et al., 2012; Bernier-Latmani et al., 2015). In addition, there is a close relationship between the gut microbiota and the immune system (Haak et al., 2018; Wiersinga and van der Poll, 2022). The gut microbiota interacts intricately with the host’s immune system, influencing immune responses through interactions with immune cells. Specific components of the microbial community can activate immune cells, leading to inflammatory responses and immune reactions closely associated with the occurrence and progression of arterial embolism and thrombosis. Notably, the rampant and indiscriminate use of antibiotics has resulted in heightened resistance to antimicrobial drugs, which in turn disrupts the gut microbiota of the host organism (Dorobanțu et al., 2022; Păduraru et al., 2022). It is pertinent to consider whether these antibiotics could impact the 11 gut microorganisms identified in the study, potentially influencing thrombogenesis.

Our MR study offers several advantages. First, we examined the causal effect of each biological level of gut microbiota on arterial embolism and thrombosis, ranging from the genus to the phylum level. This approach contributes to elucidating the mechanisms and interactions between the gut microbiota and host immunity, facilitating a comprehensive assessment of the influence of various bacterial traits. Additionally, the genetic variants of the gut microbiota were sourced from the largest GWAS available, enhancing the credibility of our findings. However, our MR study also has limitations. First, while we identified causal associations from exposure to outcomes, the magnitude of these associations may not be accurately gauged. Further research is necessary to validate these findings. Second, the use of multiple statistical corrections may have been overly stringent and conservative, potentially leading to the oversight of bacterial traits with a causal association with sepsis. Therefore, given biological plausibility, we did not consider multiple testing results. Third, although the majority of participants in our study with gut microbiota data were of European descent, a small portion of microbiological data originated from other racial backgrounds, potentially confounding our estimates. Fourth, we used a less strict threshold (*p* < 1 × 10^−5^) for horizontal pleiotropy examination and sensitivity analysis. While this allowed for the identification of a broader range of associations, it also increased the risk of detecting false positives. *Fifth, this study did not collect information on the stage, duration, and severity of* arterial embolism and thrombosis to further analyze the effect of gut microbes on different stages of arterial embolism and thrombosis. Sixth, we could not rule out the possibility of residual confounding by some uncontrolled factors (e.g., viruses and fungi), which may bias the gut microbiota and influence arterial embolism and thrombosis.

Ultimately, our study is confined to data analysis, lacking direct experimentation in animal models and validation in patient cohorts. Further confirmation of our conclusions through subsequent experiments is imperative. In summary, our study reaffirms the causal effects of gut microbiota on the risk of arterial embolism and thrombosis. Three bacterial taxa—the genera Catenibacterium, Dialister, and Odoribacter—identified in this study may be causally linked to the risk of arterial embolism and thrombosis. These findings may offer insights into the pathogenesis and potential novel treatments for arterial embolism and thrombosis.

## Data availability statement

Publicly available datasets were analyzed in this study. This data can be found at: Finngen R10 (https://r10.finngen.fi/); MiBioGen (https://mibiogen.gcc.rug.nl/) for releasing the GWAS summary statistics.

## Author contributions

Y-BS: Data curation, Formal analysis, Investigation, Methodology, Writing – original draft, Writing – review & editing. H-LD: Data curation, Formal analysis, Investigation, Methodology, Supervision, Writing – original draft. W-KC: Data curation, Formal analysis, Investigation, Writing – original draft, Writing – review & editing. YZ: Data curation, Formal analysis, Investigation, Methodology, Writing – review & editing. H-JJ: Data curation, Investigation, Methodology, Writing – original draft. J-KL: Conceptualization, Data curation, Investigation, Methodology, Writing – original draft. SY: Investigation, Methodology, Supervision, Validation, Writing – review & editing.
